# The Blood-Brain Barrier: Implications for Experimental Cancer Therapeutics

**DOI:** 10.1146/annurev-cancerbio-061421-040433

**Published:** 2023-01-25

**Authors:** Joelle P. Straehla, David A. Reardon, Patrick Y. Wen, Nathalie Y.R. Agar

**Affiliations:** 1 Department of Pediatric Oncology, Dana-Farber Cancer Institute, Harvard Medical School, Boston, Massachusetts, USA; 2 Division of Hematology/Oncology, Boston Children’s Hospital, Harvard Medical School, Boston, Massachusetts, USA; 3 Koch Institute for Integrative Cancer Research at MIT, Cambridge, Massachusetts, USA; 4 Center for Neuro-Oncology, Dana-Farber Cancer Institute, Harvard Medical School, Boston, Massachusetts, USA; 5 Department of Internal Medicine, Brigham and Women’s Hospital, Harvard Medical School, Boston, Massachusetts, USA; 6 Department of Neurology, Brigham and Women’s Hospital, Harvard Medical School, Boston, Massachusetts, USA; 7 Department of Neurosurgery and Department of Radiology, Brigham and Women’s Hospital, Harvard Medical School, Boston, Massachusetts, USA; 8 Department of Cancer Biology, Dana-Farber Cancer Institute, Harvard Medical School, Boston, Massachusetts, USA

**Keywords:** blood-brain barrier, central nervous system tumors, therapeutic development, drug delivery, pharmacokinetics, pharmacodynamics

## Abstract

The blood-brain barrier is critically important for the treatment of both primary and metastatic cancers of the central nervous system (CNS). Clinical outcomes for patients with primary CNS tumors are poor and have not significantly improved in decades. As treatments for patients with extracranial solid tumors improve, the incidence of CNS metastases is on the rise due to suboptimal CNS exposure of otherwise systemically active agents. Despite state-of-the art surgical care and increasingly precise radiation therapy, clinical progress is limited by the ability to deliver an effective dose of a therapeutic agent to all cancerous cells. Given the tremendous heterogeneity of CNS cancers, both across cancer subtypes and within a single tumor, and the range of diverse therapies under investigation, a nuanced examination of CNS drug exposure is needed. With a shared goal, common vocabulary, and interdisciplinary collaboration, the field is poised for renewed progress in the treatment of CNS cancers.

## INTRODUCTION

Primary and metastatic central nervous system (CNS) cancers are common and challenging to treat. Over 85,000 primary brain tumors are diagnosed each year in the United States (Ostrom et al. 2021). Metastases to the brain from extracranial solid tumors are even more common, accounting for up to 170,000 diagnoses in the United States each year and impacting up to 30% of adult and 10% of pediatric cancer patients (Lamba et al. 2021). The incidence of metastatic tumors to CNS is increasing as targeted therapies extend survival of patients with cancer (Frisk et al. 2012, Nayar et al. 2017, Achrol et al. 2019). Regardless of age or underlying cancer type, patients with metastatic CNS cancer have poor outcomes, with a life expectancy of six months on average (Stelzer 2013).

Cancers of the CNS are extremely heterogeneous but unified in one aspect: Outcomes have improved only slightly over the last 40 years (Miller et al. 2021) compared with continued incremental improvement in outcomes for the majority of cancers outside the CNS (Arnold et al. 2019). Strikingly, malignant CNS tumors have surpassed leukemia to become the leading cause of cancer death in children (Ostrom et al. 2021), a trend attributed to improvement in outcomes for leukemia and stagnation in outcomes for malignant CNS tumors. Even for patients with nonmalignant CNS tumors and excellent long-term survival, morbidity due to cancer-directed therapy represents a significant burden to patients and society at large.

This review focuses on the critical role of the blood-brain barrier (BBB) for the clinical treatment of both primary and metastatic CNS tumors, highlighting key findings from clinical practice and translational research. Further, we underscore the need to critically evaluate the role of the BBB in relevant clinical contexts and to share common terminology and context when discussing therapeutics under evaluation for patients with CNS cancer. Finally, we review three key clinical questions that highlight the opportunity for lab-based researchers to collaborate with clinicians to elucidate the role of the BBB and move the field of CNS therapeutics forward.

## THE BLOOD-BRAIN BARRIER IS CRITICALLY IMPORTANT FOR THE TREATMENT OF CNS TUMORS

The BBB is a complex interface between the systemic circulation and the CNS. While we utilize the well-known abbreviation “BBB” throughout this review, the term “neurovascular unit” (NVU) is a more inclusive term reflecting the dynamic role of this highly regulated interface in numerous aspects of homeostasis including the transport of molecules between compartments.

The anatomy and physiology of the BBB and the blood-tumor barrier have been well reviewed with respect to both the native function and important considerations for CNS cancers (Abbott et al. 2006, Abbott & Friedman 2012, Obermeier et al. 2013, Daneman & Prat 2015, Banks 2016, Langen et al. 2019, Arvanitis et al. 2020, Zhou et al. 2020), and we refer readers to these works for an in-depth look at the current state of the field. As it pertains to CNS therapeutic development, there are several differences important to briefly mention between the BBB and systemic capillary beds. First, the presence of tight cell junctions and basement membranes combined with a lack of fenestrations severely limit the passage of many molecules, including therapeutics, through the BBB (Hawkins & Davis 2005). Second, the BBB is characterized by a highly controlled network of influx and efflux transporters that allow for the rapid exchange of necessary nutrients (Banks 2016). In particular, several transporters from the ATP-binding cassette (*ABC*) gene family can actively pump therapeutic compounds out of the CNS (Löscher & Potschka 2005). Third, the multicellular structure of the BBB underlies the dynamic function of the BBB. As neuroscience techniques have advanced, individual contributions of endothelial cells, pericytes, astrocytes, neurons, andmicroglia to BBB integrity have been described. The BBB is heterogeneous anatomically, with regional differences in cellular composition and function (Winkler et al. 2013, Nyúl-Tóth et al. 2016, Hase et al. 2019, Bernier et al. 2021). Sex- and age-related differences (Bake et al. 2009, Sandoval & Witt 2011, Yamazaki et al. 2016, Kövesdi et al. 2020) and circadian fluctuations (Zhang et al. 2018, Walker et al. 2021, Furtado et al. 2022) have also been reported in preclinical models. In addition to the BBB or NVU, there are unique barriers between blood and cerebrospinal fluid (Redzic et al. 2005, Johanson et al. 2011, Liddelow 2015, Dani et al. 2021). These examples encompass only a portion of the complex vascular interfaces of the CNS and serve to remind the research community that therapeutic delivery to the CNS requires a nuanced approach.

For clinicians, the critical importance of the BBB for therapy has been made clear through patient care. One of the earliest examples of the CNS as a sanctuary site requiring a distinctive treatment approach is pediatric acute lymphoblastic leukemia. By the early 1970s, this once fatal disease had a complete remission rate of 94% with multiagent systemic chemotherapy, but CNS relapse was common and deadly. In a landmark study, Aur et al. (1972) showed that increasing the intensity of chemotherapy did not prevent CNS relapse, but the incorporation of craniospinal radiation was highly effective. Based on the dramatic improvement in overall survival, CNS-directed therapy is now standard for all patients with acute lymphoblastic leukemia, comprising intrathecal chemotherapy for all patients and radiation in select cases (Richards et al. 2013). Another vignette highlighting the importance of the BBB is the management of brain metastases in human epidermal growth factor receptor 2 (HER2)-positive breast cancer. Breast cancer is one of the most common cancers that metastasizes to the CNS, and patients with HER2-positive disease have an increased risk of developing CNS metastases. The clinical implementation of trastuzumab, a monoclonal antibody directed at HER2, in the adjuvant setting was highly effective in controlling metastases outside the CNS but did not decrease the incidence of CNS metastases (Bendell et al. 2003, Leyland-Jones 2009).

### Fundamental Considerations for the Treatment of CNS Cancers

In current standard-of-care therapy, patients with CNS cancers are treated with some combination of neurosurgery, radiation, and systemic therapies, although emerging technologies are of great interest to both clinicians and patients. Each of these fundamental treatment strategies has key advantages and limitations (Table 1). Upon initial presentation with a CNS cancer, neurosurgical evaluation is critical for both symptomatic treatment (e.g., diversion of cerebrospinal fluid for patients with elevated intracranial pressure) and obtaining diagnostic tissue, generally concurrent with tumor resection. Maximal safe surgical resection is the mainstay of initial treatment for the majority of patients presenting with CNS cancers, and the extent of surgical resection has been correlated with improved survival for many patients with primary CNS tumors (Lacroix et al. 2001, Cairncross et al. 2006, Brown et al. 2016) as well as select patients with accessible CNS metastases (Patchell et al. 1990). Radiation similarly plays a vital role in the treatment of CNS cancers, with indications ranging from consolidation after a surgical resection to primary local control in select CNS cancers not amenable for upfront surgical intervention. Radiation therapy is highly nuanced, with multiple treatment modalities that can be modulated to provide precise photon or charged-particle radiation to a well-defined region of the brain. The role of radiation therapy has been established in many primary and metastatic CNS tumors (Sulman et al. 2017, DeNunzio & Yock 2020, Vogelbaum et al. 2022).

**Figure 1:**
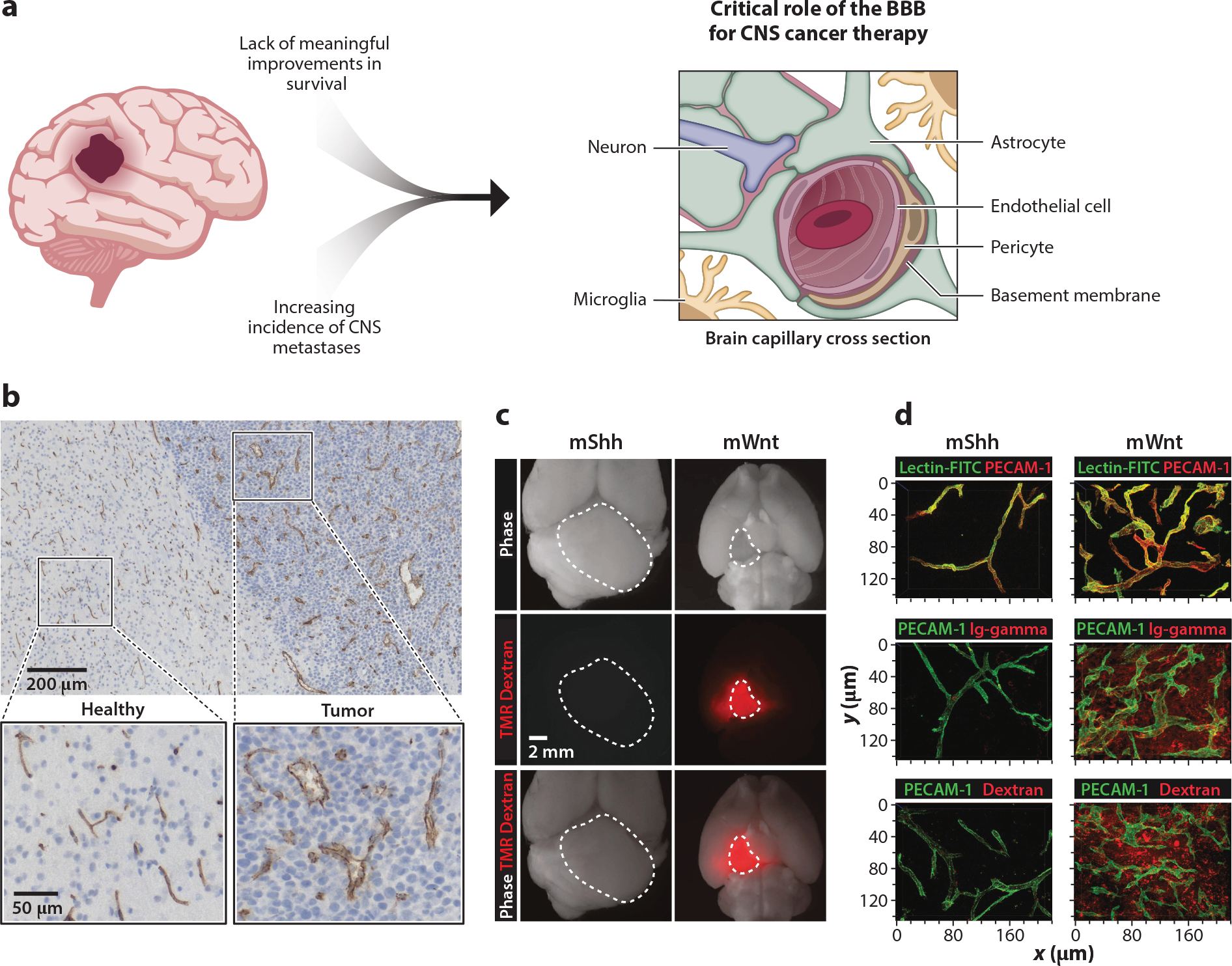
(*a*) CNS cancers are challenging to treat and the BBB plays a critical role. Key components of the BBB are labeled in a brain capillary cross section. (*b*) Brain capillaries stained with CD31 in glioblastoma tumor and surrounding healthy brain highlight high vascular density throughout, but abnormal blood vessels within the tumor bed. Panel *b* adapted from Liu et al. (2013). (*c*) Genetically engineered models of medulloblastoma showcase a genotype-phenotype relationship wherein mShh tumors exhibit morphologically normal vasculature without dextran extravasation whereas mWnt tumors exhibit abnormal vasculature with high permeability to dextran. (*d*) Tumor vessels are highlighted by systemically injected lectin-FITC and antibody staining for PECAM-1; leakage of endogenous Ig-gamma and systemically injected TMR dextran are higher in mWnt tumors than in mShh tumors. Panels *c* and *d* adapted with permission from Phoenix et al. (2016). Abbreviations: BBB, blood-brain barrier; FITC, fluorescein isothiocyanate; Ig-gamma, immunoglobulin G; mShh, Sonic Hedgehog mutant protein; mWnt, Wingless mutant protein; PECAM-1, platelet endothelial cell adhesion molecule 1; TMR, tetramethylrhodamine.

Both surgery and radiation therapy provide symptom control and can extend disease-free survival, but malignant CNS cancers—especially high-grade tumors or metastases—are rarely cured. Surgery and radiation each act as local control measures and despite marked technical advances remain limited by local toxicity and or late effects, which can be highly morbid depending on the developmental age of the patient and region(s) of the brain effected. Inevitably, cancer cells are present beyond the surgical margin or radiation field; thus, for definitive treatment, all cancer cells must be addressed over time. For these reasons, there is intense focus on utilizing systemic therapies such as chemotherapy, immunotherapy, and molecularly targeted therapies in order to improve outcomes. Brain capillaries are extremely dense with over 100 billion vessels, and it is estimated that each neuron is only 10–20 micrometers from the nearest capillary (Schlageter et al. 1999, Nicholson 2001). This level of vascular coverage within the brain highlights another important aspect of the BBB in CNS cancer therapy: delivery through the BBB is the only way to expose all cancer cells to a therapeutic agent, including those cells that may be distant from the primary tumor (Figure 1). Notably, there are many emerging technologies that seek to increase delivery across the BBB or circumvent the BBB in some cases (see further discussion in the sidebar titled New Technologies to Enhance CNS Exposure).

### Heterogeneity of the Blood-Brain Barrier Impacts Therapy

Primary tumors of the CNS are highly heterogeneous, comprised of over 100 histologically distinct tumors that can occur throughout the brain, spinal cord, choroid plexus, and meninges (Louis et al. 2021, Ostrom et al. 2021). Classification takes into account histologic features, anatomic location, and, increasingly, the presence or absence of certain molecular and genetic features (Louis et al. 2016). The anatomic site of a tumor is critically important for treatment and prognosis, as surgical resection remains a mainstay of therapy. Within individual tumors, single-cell transcriptomic methods have been utilized to explore cellular heterogeneity, which can drive therapy response (Darmanis et al. 2017, Filbin et al. 2018, Hovestadt et al. 2019, Neftel et al. 2019, Gojo et al. 2020). Analogous work focusing on brain vasculature has begun to shed light on the heterogeneity of tumor-associated endothelial cells (Teuwen et al. 2021, Xie et al. 2021, Garcia et al. 2022). Together, data from emerging single cell technologies emphasize what has been anecdotally accepted for decades: The microenvironment of CNS cancers is influenced by both tumor biology and vascular biology.

The functional implications of heterogenous tumor vasculature are not well understood, but for some CNS cancers, a genotype-phenotype relationship has been thoroughly investigated with Implications for patient care. For example, Phoenix et al. (2016) used genetically engineered mouse models to show that medulloblastomas driven by alterations in the wingless (Wnt) pathway are characterized by a compromised BBB, leading to increased exposure to systemically delivered chemotherapies and tumor response. In contrast, medulloblastomas driven by alterations in the sonic hedgehog (Shh) pathway have relatively preserved BBB integrity and are more resistant to chemotherapy (Figure 1). These preclinical observations are in line with patient outcomes, as patients with WNT-driven medulloblastoma have improved survival over those with SHH-driven tumors when receiving equivalent therapy (Gajjar et al. 2021), although other factors may also be involved. This compelling work highlights the need for additional investigations of BBB integrity and the impact on drug delivery for other primary and metastatic CNS cancers.

In the case of metastatic CNS cancers, heterogeneity of the BBB has also been well documented based on underlying tumor biology, the location of metastasis (e.g., parenchymal versus leptomeningeal), and routes of CNS spread after initial metastatic event (Carmeliet & Jain 2011, Wang et al. 2018, Achrol et al. 2019, Arvanitis et al. 2020). There are many ongoing research studies within specific cancer models seeking to link molecular cancer features to BBB phenotype, and an improved understanding of this field will be impactful for development of new therapies. Differences between the BBB in metastases and primary brain tumors are not only significant from the lens of biology and physiology but also relevant for the development of clinical trials (see the sidebar titled Key Differences in the Blood-Brain Barrier of Metastases Versus Primary CNS Tumors).

## THE NEED TO REDEFINE “CNS-PENETRANT” USING CONSISTENT, QUANTITATIVE MEASURES

The use of the term “CNS-penetrant” to describe therapeutic agents’ ability to cross the BBB is pervasive in neuro-oncology, but the ability to cross the BBB does not determine whether an agent has clinical utility. There is no accepted definition of “CNS-penetrant,” and the term implies that delivery to the CNS is an all-or-nothing venture. Furthermore, the notion that a CNS-penetrant agent will be clinically effective is grossly oversimplified, as the ability of a therapeutic agent to cross the BBB must be considered along with its distribution, clearance, and metabolism and ultimately paired with pharmacodynamic endpoints to determine whether the intended mechanism of action took place at the tissue of interest. Rather than perpetuate the use of an inarticulate term, we propose consistent reporting of well-defined, quantitative CNS pharmacokinetic measures and pairing these data with individualized pharmacodynamic measures in order to advance new therapies to the clinic for patients with CNS cancer.

### A Shift to Define CNS Exposure in Quantitative Terms

The classical pharmacokinetic principles of absorption, distribution, metabolism, and elimination combine to determine the exposure of a therapeutic agent at a given site. These principles are influenced not only by innate drug properties and administration but also by host factors, and in the CNS there are several additional factors that must be considered. The principles of CNS pharmacokinetics have been expertly reviewed elsewhere (Motl et al. 2006, Muldoon et al. 2007, de Lange 2013, Di et al. 2013, Loryan et al. 2015, Warren 2018), and we refer readers to these works for deeper study. There are several key terms defined in Table 2 that are useful in assessing the CNS exposure of a drug at specific sites of interest. The rate of brain permeability (*P*_app_) describes the ability of an agent to cross the BBB, but may be misleading if the drug is rapidly metabolized or cleared. Importantly, the unbound concentration of a drug is the most pertinent for pharmacologic activity for most small-molecule therapies and should be assessed at the desired site of action. The unbound brain-to-plasma ratio is also important, as this metric considers not only the potential of a drug to cross the BBB but also the distribution equilibrium between the blood and brain compartments. And while commonly reported, the total brain-to-plasma ratio is rarely clinically relevant, as the total drug concentration may be primarily driven by nonspecific binding to proteins and lipids. In addition to considering the concentration of unbound drug, pharmacokinetic models attempt to account for clearance (through either efflux or metabolism) through calculated measures such as the volume of distribution (*V*_u,brain_) and the calculated octanol-water coefficient (*cLogP*).

To begin to define therapeutic CNS exposure, and hence provide the most effective way to develop a drug for the clinic, researchers must not look at pharmacokinetic measures in isolation but rather consider them together with drug mechanism of action, potency, and expected toxicities. For example, the chemotherapeutic thiotepa has high apparent brain permeability (*P*_app_) with similar unbound drug levels in blood and cerebrospinal fluid (*K*_p,uu_ ~ 1) but is rapidly metabolized with a half-life of less than 2 hours (Heideman et al. 1989, 1993). Thus, exposure in brain tissue is short lived, but high peak unbound concentration in tumor (*C*_*b,u*_) can be achieved. These data, combined with knowledge of the mechanism of action (DNAalkylator) and dose-limitingtoxicity (myelosuppression), have led to the effective implementation of thiotepa as a conditioning agent for autologous stem cell transplants for select CNS cancers (Dunkel et al. 1998). Conversely, the pan-PI3K (phosphatidylinositol 3-kinase) inhibitor buparlisib has a high unbound tumor-to-plasma ratio, but when pharmacokinetic measures were paired with molecular response the agent was not potent enough to effectively inhibit the PI3K pathway and elicit clinical responses, highlighting the need to pair CNS exposure with pharmacodynamic effects and measures of response (Wen et al.2019b).

**Figure 2:**
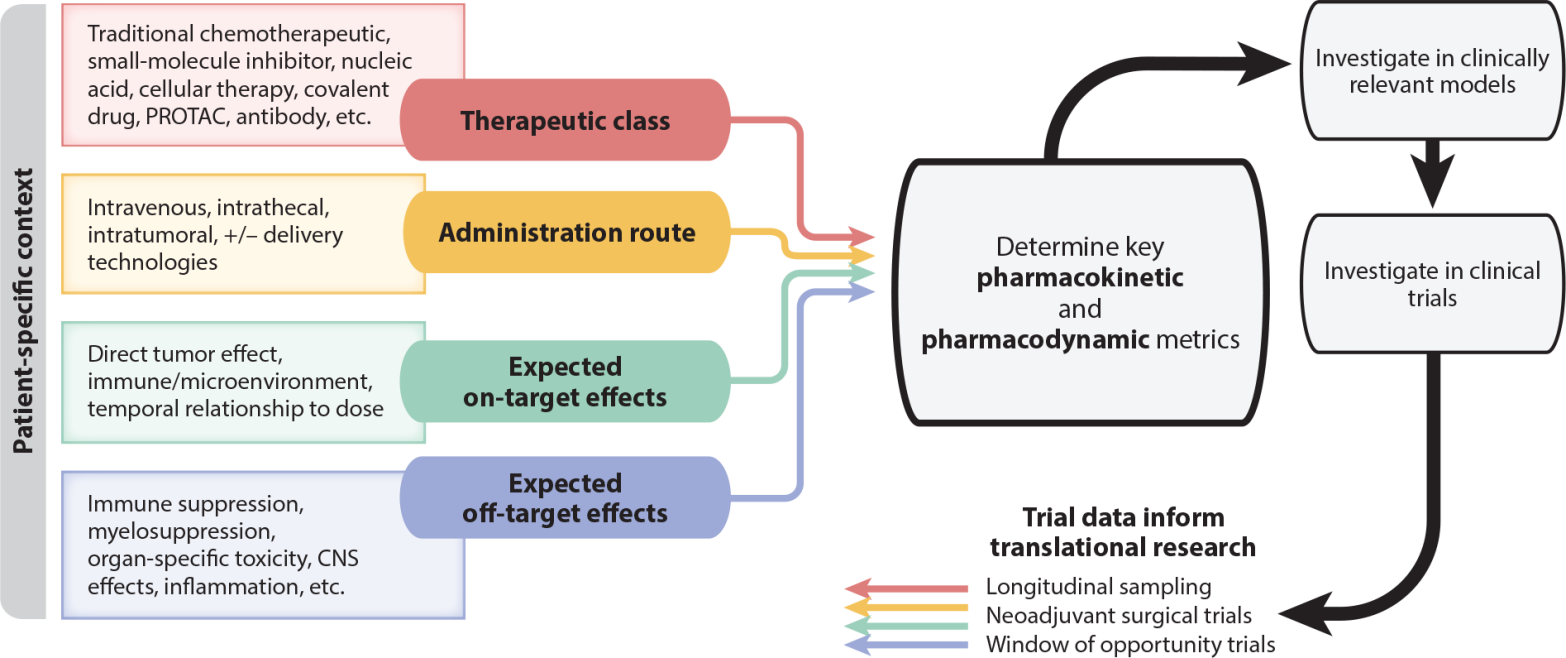
Integrative prioritization criteria for new therapeutic agents for CNS cancers, emphasizing the need to examine multiple factors within a patient specific context. After clinical trial investigation, trial data should feed back to inform specific aspects of translational research. Abbreviation: PROTAC, proteolysis-targeting chimera.

In prioritizing compounds for therapeutic development, decisions based solely on one pharmacokinetic measure may easily lead to poor efficacy due to inadequate exposure and a poor balance of on- and off-target effects. Conversely, requiring that a therapeutic agent meet overly stringent pharmacokinetic criteria may lead to missed opportunities. We propose that CNS exposure be discussed using quantitative pharmacokinetic measures within the context of a patient population of interest and with a clear understanding of both the potency of the agent and anticipated on- and off-target effects (Figure 2). Readers are referred to the sidebar titled Key Differences in the Blood-Brain Barrier of Metastases Versus Primary CNS Tumors for additional discussion of effective therapeutic exposure relevant to select patient populations.

### Not a One-Size-Fits-All Approach: New Therapies Require New Evaluation Tools

Traditional drug discovery pipelines have focused on small molecules, but emerging therapeutic agents will require special considerations related to both pharmacokinetic and pharmacodynamic properties.

For example, there is renewed interest in intrathecal therapy for the treatment of CNS cancers, particularly those with leptomeningeal spread (Nayar et al. 2017, Wang et al. 2018). In the case of intrathecal administration, measures like unbound brain-to-plasma ratio (*K*_p,uu_) are not relevant, but instead the concentration of unbound drug in cerebrospinal fluid (*C*_CSF,u_) over time would be most representative of exposure at the site of action. In the same vein, selection criteria of drugs for intrathecal delivery should be different from those used for systemic delivery, with particular attention paid to expected site-specific toxicity. For example, the inadvertent administration of the vinca alkaloid vincristine into the CSF has resulted in fatalities due to severe neurotoxicity (Dyer 2001, Chotsampancharoen et al. 2016). Other agents, such as the antimetabolites cytarabine and methotrexate, are routinely used for intrathecal treatment of hematological malignancies, although each has a narrow therapeutic window (Kwong et al. 2009, Clement & Holle 2017).

From a practical standpoint, the ability to quantify drug concentration relies on reproducible analytical chemistry techniques. For most small-molecule therapeutics, high-performance liquid chromatography and liquid chromatography-mass spectrometry are commonly employed and generally highly accurate. In contrast, emerging small-molecule therapeutic approaches like covalent binders or proteolysis-targeting chimeras (PROTACs) can be difficult to characterize with conventional analytical techniques. Specifically, the abilities to determine and interpret unbound drug fraction (given that protein binding is intrinsic to the mechanism of action) and to accurately classify and quantify metabolites are two challenges being actively investigated in these fields (Singh et al. 2011, Pike et al. 2020). Nucleic acids are a class of biologic therapeutics with great potential for targeting of genetic alterations or augmenting immune responses, but they also are confronted by delivery challenges and are difficult to quantify at the target site. Because of their labile nature and short half-life in circulation, delivery is generally augmented through viral or nonviral vectors, but detection and quantification of the vector are not necessarily reflective of exposure to the nucleic acid cargo (Kulkarni et al. 2021). Other biologic therapies such as monoclonal antibodies and cytokines generally have higher molecular weights, and their mechanisms of action are often linked to binding affinity, which also impacts distribution and clearance. Quantification of biologics in tissue can be enhanced with the use of enzyme-linked immunosorbent assays, and proteins in particular may be more amenable to chemical modification and imaging-based detection methods than small molecules (Zhao et al. 2012). In addition to the wide-ranging therapeutic classes that are being investigated for CNS cancer therapy, new technologies are being implemented that add another layer of complexity to CNS pharmacokinetic measurement (see the sidebar titled New Technologies to Enhance CNS Exposure).

As the ultimate goal of CNS cancer therapy is to exert the intended mechanism of action at the site of interest, the development of quantitative pharmacodynamic endpoints is essential to guide therapy development. In the case of targeted small molecules, a molecular pharmacodynamic measure may be straightforward, such as reduced phosphorylation of a downstream protein after treatment with an inhibitor. For example, measuring extracellular signal-regulated kinase phosphorylation in peripheral blood monocytes has been an effective strategy to define effective dosing regimens for MEK (mitogen-activated protein kinase kinase) inhibitors in extracranial solid tumors (Jamieson et al. 2016). For covalent drugs and PROTACs, the ideal pharmacodynamic measure may be a downstream effect (e.g., downregulation of gene expression within a set biologic pathway). For biologics, especially those leading to activation of the immune system, there may be temporal or spatial dissociation between the pharmacokinetic and pharmacodynamic parameters of interest, complicating both preclinical and clinical evaluation of effective exposure. To characterize the response of novel agents, longitudinal assessments can be very powerful. Clinical trials incorporating window of opportunity designs, neoadjuvant agents with surgical endpoints, and longitudinal surgical sampling can provide valuable tumor tissue to assess response.

### Spatial Drug Imaging Provides Another Level of Detail

Historically, drug concentration has been measured in body fluids (plasma, cerebrospinal fluid, etc.) or tissue homogenate, and there continues to be an important role for these metrics, as discussed above. The use of spatial drug imaging can supplement traditional pharmacokinetic parameters and complement with other spatial techniques such as radiologic imaging, immunohistochemistry, and emerging spatial profiling technologies. There are many benefits of spatial imaging, especially as it relates to tumor heterogeneity and the relationship of drug concentration to vasculature or other anatomic/morphologic features. The goal of spatial drug imaging is to quantitatively describe drug distribution within tissues with high fidelity and correlate with imaging data. Drug visualization can be via radiolabeling, fluorescent labeling, or label-free methods based on mass spectrometry (MS). Radiolabeling of therapeutic compounds can allow for visualization with positron emission tomography (PET) scanning and provide highly sensitive drug detection while maintaining compatibility with clinical anatomy scans as well as radiotracers for pharmacodynamic studies (e.g., [^18^F]-fluorodeoxyglucose, which is used to visualize glucose uptake and phosphorylation) (Workman et al. 2006, Matthews et al. 2012). However, radiolabeling is time and resource intensive, requiring specialized equipment to generate a labeled compound and regulatory approval, reducing its use at early stages of drug development, despite its potential. Fluorescent labeling is much more accessible and is commonly used in preclinical settings to study the distribution of biologics such as antibodies. However, the incorporation of fluorophores can modify the chemical properties of an agent (as opposed to radiolabeling), and fluorescence signals are prone to photobleaching and attenuation in deep tissues, which must be accounted for in quantitative analyses (Azhdarinia et al. 2012, Wüstner et al. 2012). For both radiolabeling- and fluorescence-based assays, active metabolites may not retain the label, limiting the utility for some agents. Despite these limitations, there are clear advantages to noninvasive imaging in the setting of CNS cancers. For example, quantitative CNS pharmacokinetics of the ATM (ataxia telangiectasia mutated) inhibitor AZD1390 were determined in healthy adults by administering microgram doses of a radiolabeled version and performing high-resolution PET and magnetic resonance imaging. In this case, metabolites did retain labeling, and concurrent blood collection allowed for sophisticated modeling with metabolite correction to obtain clinically relevant measures of CNS drug exposure that will inform future clinical trials (Jucaite et al. 2021).

**Figure 3:**
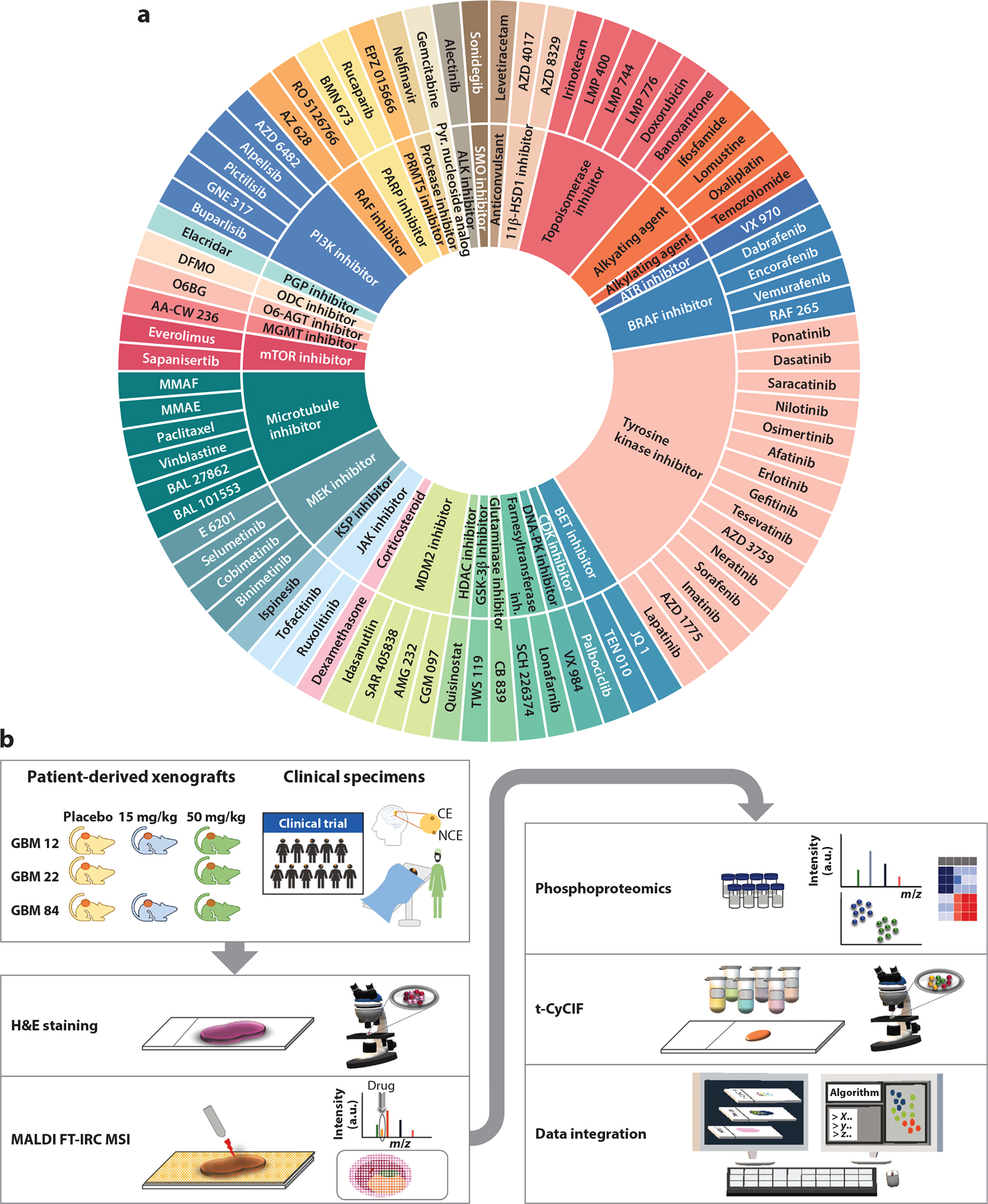
(*a*) Therapeutic compounds that have been imaged using spatial drug imaging (*outer ring*) are classified by the mechanism of action (*inner ring*); technical references for each compound can be found in Supplemental Table 1. (*b*) Workflow for assessing drug distribution and response in patient-derived xenografts and clinical patient samples in parallel. Panel *b* adapted from Lopez et al. (2022). Abbreviations: CE, contrast enhancing; DFMO, difluoromethylornithine; GBM, glioblastoma multiforme; H&E, hematoxylin and eosin; inh., inhibitor; MALDI FT-ICR MSI, matrix-assisted laser desorption/ionization Fourier transform ion cyclotron resonance mass spectrometry imaging; MMAE/F, monomethyl auristatin E/F; NCE, non–contrast enhancing; ODC, ornithine decarboxylase; PGP, P-glycoprotein; pyr., pyrimidine; t-CyCIF, tissue-based cyclic immunofluorescence.

Label-free methods have gained traction in recent years, with the two most prominent being Raman spectral imaging and matrix-assisted laser desorption ionization with mass spectrometry imaging (MALDI-MSI). These techniques are now well established and have a wide range of versatility in characterizing novel agents in tissues ex vivo. Raman spectral imaging leverages molecular vibrations and can be used to track proteins, nucleic acids, lipids, carbohydrates, and a select group of drugs and their metabolites (Ling et al. 2002, El-Mashtoly et al. 2014). Easily combined with high-resolution microscopy, Raman has exquisite spatial resolution but is not broadly applicable across classes of drugs because detection is based on functional groups rather than full molecules. Quantitative analyses are limited at low signal-to-noise ratios, and imaging of large tissues is time consuming. MALDI-MSI harnesses the robust quantitative power of MS to detect thousands of compounds simultaneously without labels and can be readily applied to clinical samples, as the only sample preparation required is matrix deposition (Aichler & Walch 2015, Basu et al. 2019, Basu & Agar 2021). When first applied to spatial drug distribution, the number of compounds readily assayed was limited, but over the last two decades protocols have been developed for hundreds of compounds (Figure 3a; protocol references are shared in Supplemental Table 1). In addition, the spatial resolution of MALDI-MSI has improved dramatically, with the latest instruments resolving compounds to the submicron level. Similar to Raman imaging, MALDI-MSI capabilities expand beyond therapeutic compounds, and quantitative detection of native or exogenous lipids and a range of metabolites is possible. We and others have employed MALDI-MSI to simultaneous assess drug distribution and pharmacodynamic response in patient samples (Wen et al. 2019b, Freedman et al. 2020, Basu et al. 2021, Lopez et al. 2022). For example, by combining MS imaging with phosphoproteomics and multiplexed tissue imaging, both clinical samples and patient-derived xenograft models can be processed in parallel (Figure 3b) (Lopez et al. 2022). Using this multimodal platform, the relationship between drug exposure and tumor response can be clearly delineated and evaluated with respect to dose and schedule. Heterogeneous tissue responses in clinical samples are explained by heterogeneous drug exposure on a pixel-by-pixel basis, an important distinction that cannot be made without spatially resolved imaging of drug and molecular response markers.

## OUTSTANDING CLINICAL CHALLENGES

In addition to rigorously assessing CNS exposure of new therapies, we highlight here two critical challenges related to the BBB that should be addressed in order to effectively implement new treatments for patients with CNS cancer.

### How Do Glucocorticoids and Antiangiogenic Agents Impact Delivery of Novel Therapies?

Glucocorticoids are highly effective in managing acute cerebral edema and are often employed as a first-line treatment for patients with elevated intracranial pressure due to CNS tumors. However, the ubiquitous use of glucocorticoids in neuro-oncology presents a unique challenge given their effects on the BBB and immune function, in addition to other morbid side effects. The mechanism by which glucocorticoids decrease vasogenic edema is not fully understood, but it is hypothesized to be due to decreased permeability at the BBB through the restoration of tight junctions, based on evidence that dexamethasone and other glucocorticoids lead to a decrease in drivers and regulators of angiogenesis such as vascular endothelial growth factor (VEGF) (Liu & Goodwin 2020, Zhou et al. 2020). In addition to effects on endothelial cells, glucocorticoids are also potent anti-inflammatory agents and may impact BBB function secondary to changes in immune cell populations and decreased cytokine production (Herting et al. 2019).

Antiangiogenic agents are also commonly employed in primary CNS tumors to inhibit tumor neovasculature. The therapeutic impact of targeting vascular mechanisms has been studied rigorously in adult glioblastoma (Carmeliet & Jain 2011, Kuczynski et al. 2019, Ribatti & Pezzella 2022) but is still an emerging area of study for many other primary and metastatic CNS cancers. There are several therapeutic modalities under investigation, but small molecules targeting VEGF receptor and monoclonal antibodies targeting VEGF have been most effective to date. The anti-VEGF antibody bevacizumab received accelerated approval by the US Food and Drug Administration (FDA) in 2009 for recurrent glioblastoma based on an improvement in progression-free survival (Vredenburgh et al. 2007). Registration studies later performed in patients with newly diagnosed glioblastoma showed a very modest progression-free survival benefit (~4 months) and no difference in overall survival (Gilbert et al. 2014).

Given their stabilizing effect on the BBB, concurrent use glucocorticoids or antiangiogenic agents may decrease CNS exposure of cancer-directed therapies. For example, bevacizumab was shown to decrease vascular permeability in a cohort of patients with recurrent glioblastoma, resulting in decreased delivery of radiolabeled temozolomide as measured by PET imaging (Gerstner et al. 2020). Outside of glioblastoma, the impact of glucocorticoids and antiangiogenic agents in patients with primary tumors other than glioblastoma and in CNS metastases remains largely unexplored. Preclinical investigation of the effect of these agents on both tumor and vascular cells across primary and metastatic tumors, including timing and dose dependence of biologic and functional changes, would be impactful. In the preclinical setting, determining the time-dependent effect of antiangiogenic therapies on vascular physiology may help identify promising combination therapies and provide a roadmap for staggered dosing.

### What Is the Impact of the Blood-Brain Barrier on CNS Cancer Immunotherapy?

Immunotherapy has transformed the treatment landscape for many cancers but has not yet had a major impact for patients with primary CNS cancer. For some patients with CNS metastases from melanoma or non-small-cell lung cancer, immunotherapy with checkpoint blockade has shown efficacy (Goldberg et al. 2020; Tawbi et al. 2018, 2021), with response rates similar to those seen in extra-CNS primary tumors of the same histology. In contrast, studies employing checkpoint blockade therapy in primary brain tumors have been largely disappointing in terms of clinical response (Omuro et al. 2018, Reardon et al. 2020, Nayak et al. 2021, Bi et al. 2022, Brastianos et al. 2022), although biologic corollary studies have shown evidence of impact on the tumor microenvironment (Schalper et al.2019, Zhao et al. 2019), and neoadjuvant dosing has led to survival benefit in recurrent glioblastoma (Cloughesy et al. 2019). In addition to immune checkpoint inhibitors (such as PD-1, PD-L1, and CTLA-4 inhibitors), there are several immunotherapies in development for CNS cancers, including vaccines (Yamanaka et al. 2005, Platten et al. 2018, Hilf et al. 2019, Keskin et al. 2019, Narita et al. 2019, Wen et al. 2019a), cellular therapies such as chimeric antigen receptor (CAR) T cells (Brown et al. 2015, Ahmed et al. 2017, O’Rourke et al. 2017, Goff et al. 2019, Majzner et al. 2022), and oncolytic virus therapy (Desjardins et al. 2018, Cloughesy et al. 2018, Lang et al. 2018, Friedman et al. 2021, van Putten et al. 2022).

There are many factors that likely contribute to the disparate outcomes for immunotherapy between extracranial and intracranial solid tumors, which have recently been reviewed in detail (Chuntova et al. 2021, Flores et al. 2022). Particularly notable for the present review is the fact that most immunotherapies evaluated in clinical trials thus far are antibodies with poor innate CNS exposure. The impact of the BBB on immune therapies is complex, as the mechanism of action of immune therapies may be spatially and temporally distinct from the administration method. In the case of CAR (chimeric antigen receptor) T cells, direct administration into tumors has been more effective than systemic administration, which suggests that improving exposure at the site of the tumor is important to improve the therapeutic window for this mode of therapy (Akhavan et al. 2019). Another complicating factor is that many patients treated with immunotherapies also receive glucocorticoids, which lead to T cell apoptosis and cytokine depletion. Sustained corticosteroid usage has been associated with blunted immune responses in patients with breast cancer brain metastases receiving immune checkpoint inhibitors (Martínez Bernal et al.2017). In preclinical studies, dexamethasone was shown to limit the clinical benefit of immune checkpoint blockade in glioblastoma in a dose-dependent manner, correlating with patient data (Iorgulescu et al. 2021).

Much work remains to be done to explore the impact of the BBB and corticosteroids on immunotherapy efficacy across the range of primary and metastatic CNS cancers. Basic researchers and clinicians should collaborate to share data from negative clinical trials to inform preclinical investigations. Going forward, remaining open-minded about new combinations, temporally staggering therapies, and implementing new drug delivery technologies will increase the chances for successful translation.

## PRECLINICAL MODELS TO EVALUATE NOVEL CNS THERAPIES

**Figure 4:**
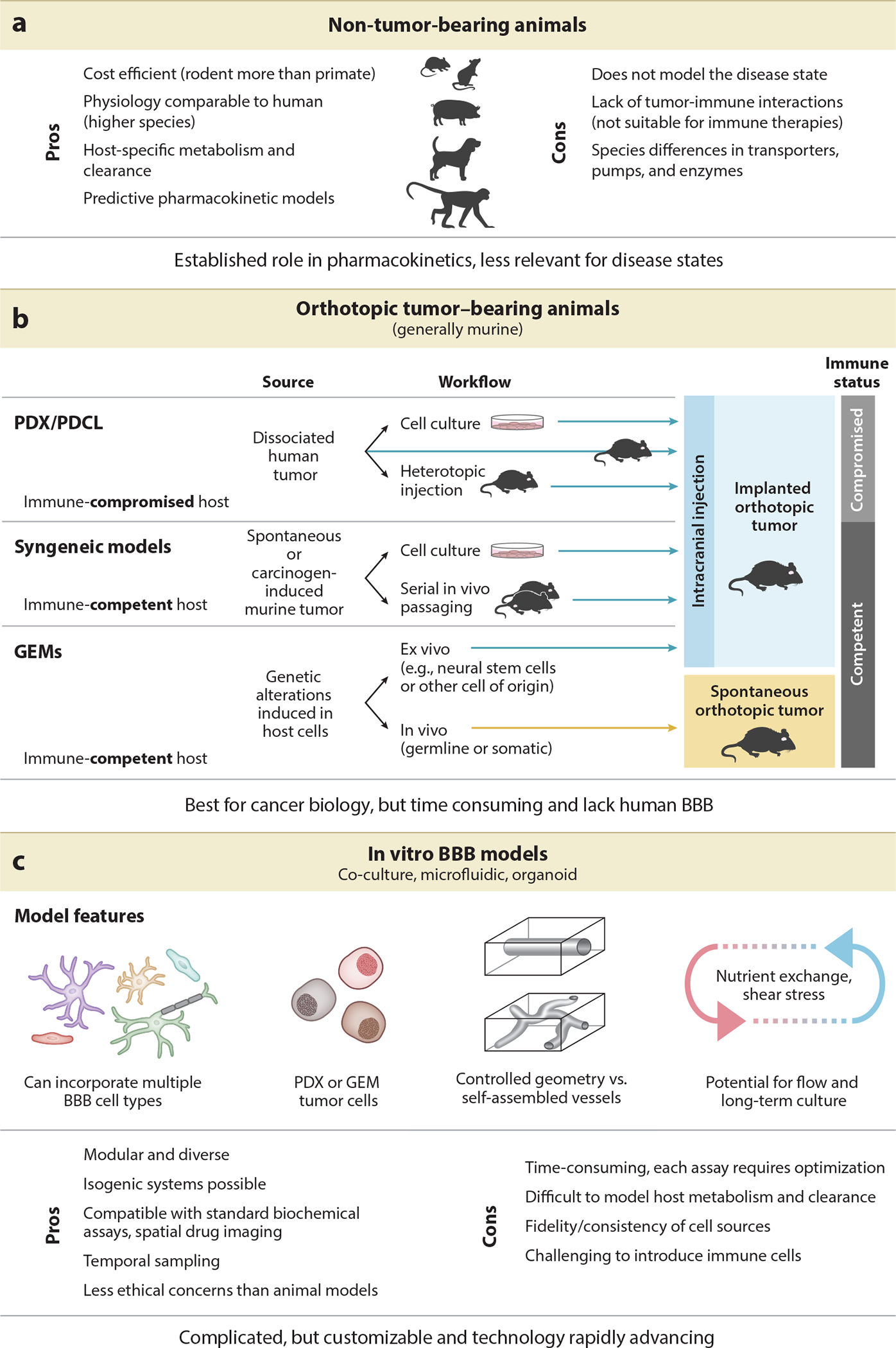
Preclinical models used to assess BBB permeability of CNS therapeutics. (*a*) Non-tumor-bearing mice are utilized to determine pharmacokinetic parameters in healthy animals. (*b*) Orthotopic tumor models can be patient-derived, syngeneic, or genetically engineered. (*c*) In vitro models are modular and diverse, with high potential for customization but limited predictive ability at this time. Abbreviations: BBB, blood-brain barrier; GEM, genetically engineered model; PDCL, patient-derived cell line; PDX, patient-derived xenograft.

There are many preclinical models that may be employed to address these questions, each with specific advantages and disadvantages with respect to drug delivery. For each therapeutic class and administration route under investigation, the choice of preclinical model(s) may be different. While not an exhaustive list, there are three major classes of BBB models that can be employed for preclinical therapeutic assessments (Figure 4).

### Non-Tumor-Bearing Animals: Employed for Robust Pharmacokinetics

After identifying a therapeutic agent with potential applications for the CNS, non-tumor-bearing rodents are typically employed for first-pass CNS pharmacokinetic parameters (Table 2) (Di et al. 2013). Murine and rat models are accessible, and protocols for dosing and sampling of body fluids/tissues are well established. If a therapy advances to the stage of submission to the FDA as an Investigational New Drug, rodent metabolic and toxicity studies are required (FDA 2021). One major advantage of this approach is that data can be quantitatively compared across large data sets for many different drugs. However, there are important physiological and biological differences between the rodent BBB and higher-order species such as pig and nonhuman primate, especially as it relates to transporters and enzymes that can impact CNS exposure (Hoshi et al. 2013, Morris et al. 2017). Pigs have been effective in modeling CNS physiology for degenerative disease (Hoffe & Holahan 2019), although they are harder to access and expensive. While the abundance of data from non-tumor-bearing animal models has led to robust predictive models of CNS pharmacokinetics (de Lange 2013, Feng et al. 2018), the relevance of these metrics for patients with CNS tumors is less clear given the heterogeneity of the BBB in states of disease. Overall, non-tumor-bearing animals, especially rodent models, should continue to be employed to determine CNS pharmacokinetic measures, but are not sufficient to predict drug exposure or pharmacodynamic impact in human CNS cancers.

### Orthotopic Xenografts and Genetically Engineered Models: Best for Cancer Biology

Human tumor xenografts have been successfully employed to study a range of cancers and can be simply defined as human cancer cells implanted into an animal host, with murine hosts being the most common. There are many nuances of xenograft models, including the source of human cells, the genetic makeup of the host, and the location of cell implantation (Hidalgo et al. 2014). One important distinction is whether cells are passaged directly from patient to host (patient-derived xenograft) or first in tissue culture conditions (patient-derived cell line). In one commonly employed technique (Carlson et al. 2011), cells are first xenografted in a heterotopic location (generally flank), passaged in vivo, and then orthotopically injected in the tissue of origin (Figure 4). In all patient-derived models, an immune-compromised host is used, which is a limitation for studying immune therapies.

Syngeneic and genetically engineered models (GEMs), by contrast, are suitable for immunotherapy evaluations as tumors are derived from host cells. Traditional syngeneic models refer to cancers that occur in the host animal, either sporadically (e.g., SMA-560, identified in VM mice) or through carcinogen induction (e.g., GL261 or CT-2A, induced in C57BL/6 mice). These models are then passaged either in cell culture or through serial passaging in hosts from an identical genetic background (Oh et al. 2014, Letchuman et al. 2022). GEM models are also syngeneic, but have a key advantage in that researchers can control the mutations or other genetic perturbations introduced. If ex vivo methods are used (e.g., lentiviral transduction or CRISPR editing of neural stem cells or other cancer cells of origin), cells are then reimplanted to generate an orthotopic model (Figure 4). Alternatively, genetic alterations can be induced using lineage specific Cre drivers, direct injection of viral or nonviral vectors, or intrauterine electroporation, resulting in spontaneous CNS tumors (Kersten et al. 2017, Robertson et al. 2019, Wei et al. 2021). Finally, it is notable that spontaneous CNS cancers do arise in some higher mammals, particularly in canines. Veterinary clinical trials in canine models of glioma have been employed to test new therapies (Groothuis et al. 1990, MacDiarmid et al. 2016, Arami et al. 2019, Boudreau et al. 2021), but the incidence of spontaneous tumors and need for specialized veterinary practices precludes widespread use of canine models for drug development. Each in vivo model described above has unique advantages with respect to cancer biology and/or immunology, but lack of a human BBB may limit the utility of these models for investigating CNS therapeutics.

### Three-Dimensional In Vitro Models: Emerging Technologies with Potential for High-Throughput Screening

There are many in vitro models of the BBB that are relevant to cancer drug development, and the reader is referred to several excellent reviews on this topic for additional details (Robertson et al. 2019, Hajal et al. 2021, Joseph et al. 2021). The primary advantage of in vitro models is modularity—each model can be designed to answer a specific question. For example, incorporating BBB cells such as endothelial cells, pericytes, and astrocytes along with patient-derived tumor cells enables investigation of tumor-vascular interactions and drug trafficking (Straehla et al. 2022). Another advantage of in vitro models is flexibility in cell source. Primary human or animal cells, induced pluripotent stem cells, and engineered cells have all been successfully incorporated into three-dimensional models; there has also been great progress in using patient-specific stem cells to generate fully isogenic models (Workman & Svendsen 2020). Three-dimensional model systems can be complex and time consuming to optimize and are highly dependent on the consistency and fidelity of cell sources; these factors currently limit their usefulness for high-throughput screening. Modeling cancer cell interactions with immune cells and neurons also remains limited at this time given the complexities of modeling the complete microenvironment including nutrient availability and clearance, although this is an active area of research. Despite these current limitations, the rapid advances in technology for modeling the BBB and the ability to incorporate relevant tumor models have the potential to improve our fundamental understanding of tumor neovasculature by probing different components of the BBB. High-fidelity in vitro models can provide orthogonal data that complements information from in vivo models, and together these can accelerate drug development for CNS cancer.

### The Way Forward

Despite the challenge of the BBB, there is reason for optimism for patients with CNS cancers. As new technologies advance, the toolkit of therapies available is ever expanding and the creative combination of therapeutic classes, delivery adjuvants, and routes of administration may well lead to highly effective therapies. In this time of increasingly complexity, it is imperative for researchers and clinical trialists to speak a common language when determining if the CNS exposure of a given therapy is likely to be effective in a specific clinical context.

To advance CNS therapeutics to the clinic, we need both a return to basics (robust and quantitative pharmacokinetic measures) and nuanced measures of pharmacodynamic response. New therapies must also be studied in realistic clinical contexts, considering the cadence of surgery, radiation, and supportive medications that patients are receiving. In particular, as the field of immunotherapy progresses, wherein response may be temporally and spatially distinct from the drug mechanism of action, the ability to longitudinally measure response is critical. To make an impact for patients with CNS cancer, we must be able to learn from each clinical trial, which will require meaningful collaboration among interdisciplinary researchers.

## Supplementary Material

Supplementary Table 1

## Figures and Tables

**Table 1:** Advantages and limitations of first-line treatment modalities utilized for CNS cancers

Treatment modality	Key advantages	Key limitations
Neurosurgery	• Immediate impact on symptoms • Extent of resection correlates with survival benefit for most tumors	• Local toxicity and late effects • Unable to address microscopic disease
Radiation	• Can address multifocal disease • Tunable based on age/location/risk	• Local toxicity and late effects • Unable to address distant disease
Chemotherapy, immunotherapy, and targeted therapy	• Potential to address all cancer cells over time • Noninvasive; dose and schedule tunable	• Requires crossing the blood-brain barrier and maintaining adequate exposure to exert mechanism of action • Heterogeneous distribution contributes to adaptive response • Often limited by systemic toxicity

**Table 2:** Key CNS pharmacokinetic properties defined

Term	Standard units	Definition
*P*_app_	cm/s	Apparent brain permeability
*C*_p,u_, *C*_b,u_, *C*_t,u_, *C*_CSF,u_	g/L	Concentration of unbound drug in plasma (p), brain (b), tumor (t), or cerebrospinal fluid (CSF)
*K*_p,uu_ = *C*_b,u_/*C*_p,u_	NA (ratio)	Unbound brain-to-plasma ratio
*K*_p,brain_ = *C*_b_/*C*_p_	NA (ratio)	Total brain-to-plasma ratio (also termed logBB)
*f*_u,p_, *f*_u,b_	NA (ratio)	Fraction unbound in plasma (p) or brain (b)
*V*_u,brain_	(mL.g)/brain	Volume of distribution in brain
*cLogP*	NA (ratio)	Calculated octanol-water partitioning coefficient

## NEW TECHNOLOGIES TO ENHANCE CNS EXPOSURE

To add to the complexity of new drug classes that are being developed for CNS and other cancers, new drug delivery technologies are also being implemented to increase CNS drug exposure. Three examples of this are convection enhanced delivery, focused ultrasound with microbubbles, and chemically engineered drug carriers. Convection-enhanced delivery utilizes catheters to directly infuse therapies into tumors (Bobo et al. 1994, Bidros et al. 2010, Tosi & Souweidane 2020), and focused ultrasound uses high-frequency oscillations of circulating microbubbles to transiently disrupt the BBB in a controlled area (Arvanitis et al. 2012, Mainprize et al. 2019). Examples of chemically engineered drug carriers include nanocarriers and polymer drug delivery systems (e.g. polymer-protein, polymer-drug, or peptide-drug conjugates), just to name a few (Liechty et al. 2010, Mitchell et al. 2021). There is great interest in combining strategies for additive effect, e.g., convection-enhanced delivery of drug-loaded nanocarriers (Kumar et al. 2019). For these techniques that bypass traditional absorption routes, distribution and clearance are key properties and should be the focus of preclinical validation in concert with pharmacodynamics and clinical response.

## KEY DIFFERENCES IN THE BLOOD-BRAIN BARRIER OF METASTASES VERSUS PRIMARY CNS TUMORS

While clinical outcomes are dismal for both primary and metastatic CNS cancers, there is a developing body of literature highlighting key differences in vasculature (Arvanitis et al. 2020). In fact, several targeted agents with low intrinsic ability to cross an intact BBB have shown efficacy against CNS metastases. For example, the BRAF inhibitor dabrafenib has limited distribution across an intact BBB (Mittapalli et al. 2013), and patients with CNS metastases were initially precluded from clinical trials but ultimately were shown to have clinically significant response rates (Long et al. 2012, Ng et al. 2022). This is not to say that CNS drug distribution is irrelevant for patients with CNS metastases—on the contrary, there is strong evidence that agents with higher CNS exposure lead to improved and more durable responses, as in the case of the ALK inhibitor alectinib outperforming crizotinib in patients with ALK-positive lung cancer and CNS metastases (Peters et al. 2017). However, the pattern of clinically meaningful responses to agents with poor CNS distribution serves to emphasize that patients with CNS metastases should not be automatically excluded from clinical trials of otherwise effective agents.

## SUMMARY POINTS

1. The blood-brain barrier (BBB) is a critical factor in the treatment of central nervous system (CNS) cancers.
2. There are important differences in the BBB for metastatic and primary CNS cancers with implications for clinical trial enrollment.
3. We propose a shift from the term “CNS penetrant” to a nuanced discussion of CNS exposure that is framed by quantitative pharmacokinetic measures.
4. There is no one-size-fits-all approach, and the efficacy of new therapies should be assessed by linking CNS exposure to pharmacodynamic response within a clinical context.
5. Spatial drug imaging provides a toolkit for simultaneously assessing pharmacokinetic and pharmacodynamic measures for a wide range of new and existing therapies.

## FUTURE ISSUES

1. The majority of therapeutic development has focused on common primary CNS cancers; additional work is needed to address heterogeneous primary and metastatic cancers.
2. The implementation of new therapies should be considered in the setting of commonly employed agents such as corticosteroids and antiangiogenics.
3. A deeper understanding of the BBB and tumor microenvironment is necessary to employ immunotherapy effectively in CNS cancers.
4. Longitudinal measures of response have the potential to accelerate the development of new therapies and should be incorporated in innovative trial designs.
